# Freckle Defect Formation near the Casting Interfaces of Directionally Solidified Superalloys

**DOI:** 10.3390/ma9110929

**Published:** 2016-11-16

**Authors:** Jianping Hong, Dexin Ma, Jun Wang, Fu Wang, Baode Sun, Anping Dong, Fei Li, Andreas Bührig-Polaczek

**Affiliations:** 1 Shanghai Key Lab of Advanced High-Temperature Materials and Precision Forming, Shanghai Jiao Tong University, Shanghai 200240, China; bdsun@sjtu.edu.cn (B.S.); apdong@sjtu.edu.cn (A.D.); lifei74@sjtu.edu.cn (F.L.); 2 Foundry Institute, RWTH Aachen University, Aachen 52056, Germany; d.ma@gi.rwth-aachen.de (D.M.); f.wang@gi.rwth-aachen.de (F.W.); sekretariat@gi.rwth-aachen.de (A.B.-P.); 3 State Key Laboratory of Metal Matrix Composites, Shanghai Jiao Tong University, Shanghai 200240, China

**Keywords:** freckles, convection resistance, interface, directional solidification

## Abstract

Freckle defects usually appear on the surface of castings and industrial ingots during the directional solidification process and most of them are located near the interface between the shell mold and superalloys. Ceramic cores create more interfaces in the directionally solidified (DS) and single crystal (SX) hollow turbine blades. In order to investigate the location of freckle occurrence in superalloys, superalloy CM247 LC was directionally solidified in an industrial-sized Bridgman furnace. Instead of ceramic cores, Alumina tubes were used inside of the casting specimens. It was found that freckles occur not only on the casting external surfaces, but also appear near the internal interfaces between the ceramic core and superalloys. Meanwhile, the size, initial position, and area of freckle were investigated in various diameters of the specimens. The initial position of the freckle chain reduces when the diameter of the rods increase. Freckle area follows a linear relationship in various diameters and the average freckle fraction is 1.1% of cross sectional area of casting specimens. The flow of liquid metal near the interfaces was stronger than that in the interdendritic region in the mushy zone, and explained why freckle tends to occur on the outer or inner surfaces of castings. This new phenomenon suggests that freckles are more likely to occur on the outer or inner surfaces of the hollow turbine blades.

## 1. Introduction

Superalloys offer excellent high temperature tensile strength, stress rupture and creep properties, fatigue strength, oxidation and corrosion resistance, and micro-structural stability at elevated temperatures 600 °C or above [1,2,3,4,5,6]. Nickel-based superalloys are the most complex and the most widely used in high temperature applications. However, because of the factors of alloy chemistry, casting conditions including solidification parameters, and casting geometry, freckles, hot cracking, low angle grain boundaries, and other solidification defects were found in directional solidified (DS) and single crystal (SX) superalloy castings. These solidification defects have a negative influence on high temperature mechanical properties, and reduce the life of aero engine and gas turbine land-based power generation [7,8,9].

Freckles are macroscopic channel segregation defects which usually appear as a long trail of equiaxed grains with a composition shift consistent with alloy segregation in a wide variety of industrial castings, such as vacuum arc remelting (VAR) and electro-slag remelting (ESR) superalloy billets, nickel-based superalloys, or specialty steel, that occur during solidification. They are presently one of the main defects encountered in the advanced casting technology of superalloys [10]. Macro-segregation such as freckles cannot be removed by post-processing, thermo-mechanical treatments, or plastic processing [11,12]. Since the 1960s, when they were linked to the failure of several military engines, freckles are considered to be unacceptable defects in industrial aerospace castings. Freckle defects are highly undesirable in critical applications because of their deleterious effect on mechanical performance and because they cause considerable economic loss.

It is generally agreed that freckles are the product of specific fluid flow patterns, known as thermosolutal convection, originating in the interdendritic liquid during solidification. This flow is driven by a density inversion occurring in the mushy zone as a result of interdendritic segregation. In a previous study, freckle occurrence was found to be dependent on three factors: alloy chemistry, casting conditions, and casting size [10]. Much research has been focused on alloy chemistry and solidification conditions. However, previous empirical knowledge and empirically determined casting parameters seem to be of little help in finding the new appropriate solidification conditions. The local thermal gradient (G), local solidification rate (R), and local solidification time (LST) have already been investigated. Currently, the general acceptant and most complete predictive criteria is the Rayleigh criterion, which combines two of the three factors influencing freckle formation, alloy chemistry and casting conditions, but not casting geometry. Since freckling results from the breakdown of a metastable equilibrium state (heavier liquid atop a lighter one), it is assumed that freckle initiation will always produce fully grown freckles.

In the present study, a variety of single crystal superalloy castings with various geometries such as specimens with multi-interfacial features were created in an industrial Bridgman furnace with a lower withdrawal rate, which is prone to freckle formation. The main goal of this research is freckle formation on the external surface of rods with a variety of diameters (cross sectional area) and freckle formation on both the external and internal surfaces of castings with multi-interfaces. Morphology and composition analysis were investigated with freckle and freckle free (Matrix) regions. Finally, the formation mechanism of freckle near the interface is discussed.

## 2. Materials and Methods

### 2.1. Selection of Superalloys

Superalloy CM247 LC was selected for the investigation of freckle formation in this work. CM247 LC is widely used as DS and SX parts in the highest temperature parts of aero-engines. They are working under high temperature and high-speed rotating conditions. Hundreds of pieces of single crystal turbine blades are assembled in a turbofan engine on airplanes. Of course, they should be the least amount of defects as possible. Unfortunately, in past research work, freckles which appeared during directional solidification were observed on the Ni-based DS and SX turbine blades. Superalloy CM247 LC is prone to freckle formation due to the content of elements such as Rhenium, Tungsten, Titanium, and so on, which is a kind of deleterious macro-segregation defect.

**Figure 1:**
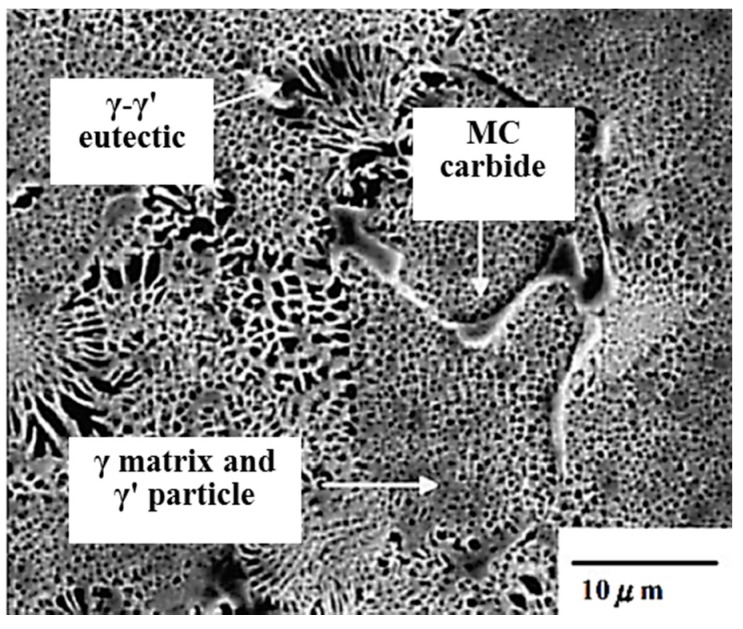
SEM micrograph showing the as-cast microstructure of the CM 247 LC superalloy.

CM247 LC is a polycrystalline cast nickel base superalloy. It is commonly produced using directional solidification techniques to improve creep rupture strength. It has exceptional high temperature strength, corrosion, and oxidation resistance. This alloy can also be obtained with hafnium additions to control the grain boundary structure; in turn, this addition prevents cracking and improves ductility during processing. CM247 LC is commonly used for blade rings and high pressure turbine blades. CM247 LC is a chemically modified superalloy derived in late 1970s from the MAR-M247 composition, specifically designed for DS blade and vane applications. The nominal composition of CM247 LC is shown in Table 1 [13]. Figure 1 [14] shows the SEM micrograph of the as-cast microstructure of CM247 LC superalloy, and γ/γ′ eutectic, MC carbide, γ matrix, and γ′ particle were detected.

### 2.2. Specimen Design

#### 2.2.1. Solid Specimens

**Figure 2:**
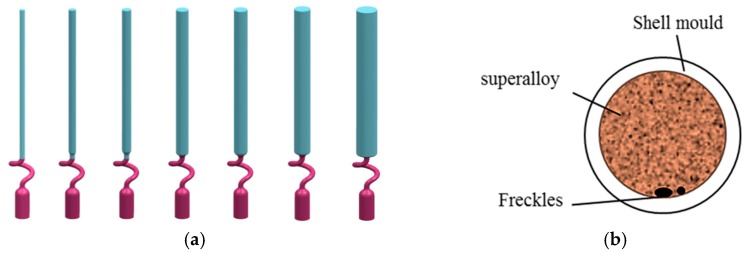
Schematic diagram of (**a**) 3D design of a series of solid rod specimens, and (**b**) possible freckle formation on the external surface.

As shown in Table 2, a series of diameters of solid rod specimens were prepared with diameters of 5 mm, 7 mm, 9 mm, 11 mm, 13 mm, 15 mm, and 19 mm. Figure 2a shows the 3D design of a series of solid rod specimens, and in the bottom of the specimen, a kind of helix (spiral crystal selector) was used for the single crystal growth, with a height of 65 mm and a 360 degree helix angle. The height of the rod specimens was 150 mm, and the cross sectional areas were 20 mm^2^, 39 mm^2^, 64 mm^2^, 95 mm^2^, 133 mm^2^, 177 mm^2^ and 283 mm^2^, respectively. The wax specimens were assembled around a central rod, in a cluster on a wax disk.

#### 2.2.2. Multi-Interface Specimens

With the increasing demand of the aerospace industry, more and more large and complex superalloy components are required for airplane engines. The complex components, especially ceramic cores, are applied in the hollow blades that cause multi-interfaces in the casting specimens.

**Figure 3:**
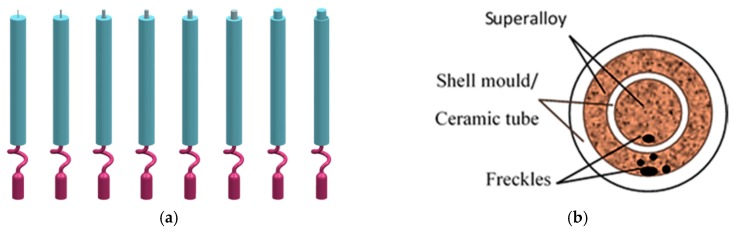
Schematic diagram of (**a**) 3D design of a series of rod specimens with multi-interfacial features and (**b**) possible freckle formation on the multi-interfaces.

In the present work, it was observed that the freckle defects occurred near the interfaces of superalloys and ceramic materials, as shown in Figure 3a. Indeed, this is the first time that the freckle defects occurred near the interface of the superalloys and ceramic core inside of casting specimens. Furthermore, due to the arrangement of the specimen on the disc in the Bridgman furnace, shown in Figure 3b, the freckle was observed on the shadow side of the specimens, which face the central rod and have a lower temperature than the heater side. A schematic diagram of the freckle defects that occur near the multi-interfaces is shown in Figure 3a; the dark parts are superalloys, and the white parts are ceramic materials (ceramic core inside of the casting or alumina shell mold outside of the casting). Because of the ceramic materials, there was more than one interface near the superalloys. The ceramic core inside of the casting provided an additional interface.

For simplification, ceramic tubes were used to replace the ceramic core inside of the castings. Ceramic tubes with various diameters were designed, to investigate their influence of interface effects to freckling (Figure 3). The size of the ceramic tube and external diameter of the specimen are shown in Table 3.

### 2.3. Procedure of Directional Solidification

**Figure 4:**
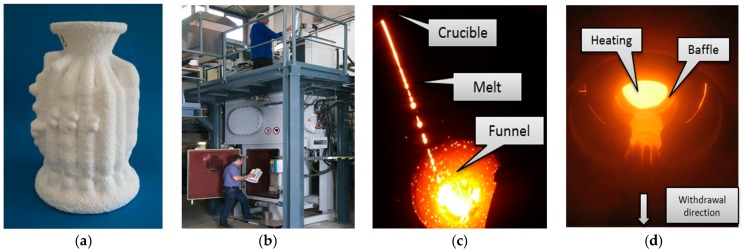
The process of directional solidification in the industrial vacuum Bridgman furnace: (**a**) Shell mold; (**b**) mold loaded in the Bridgman furnace; (**c**) melt pouring, and (**d**) directionally solidification and mold cooling.

During the casting progress, the ceramic shell mold (See Figure 4a) was placed on the copper chill plate in the Bridgman furnace shown in Figure 4b. The shell mold was preheated, poured with the superalloy melt (See Figure 4c), and then withdrawn from the heating zone through the baffle into the cooling zone, shown in Figure 4d. The employed heater and pouring temperatures were 1450 °C and 1500 °C, respectively. The single crystal solidification of the components was achieved by a grain selector. A low withdrawal velocity of *v =* 1.0 mm/min was applied to promote freckle defects.

**Figure 5:**
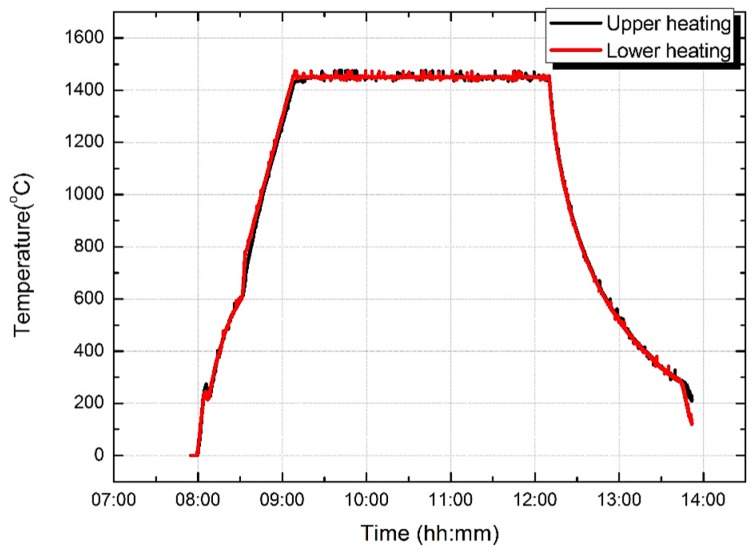
The temperature of the heating system during the solidification and cooling process in the Bridgman furnace.

**Figure 6:**
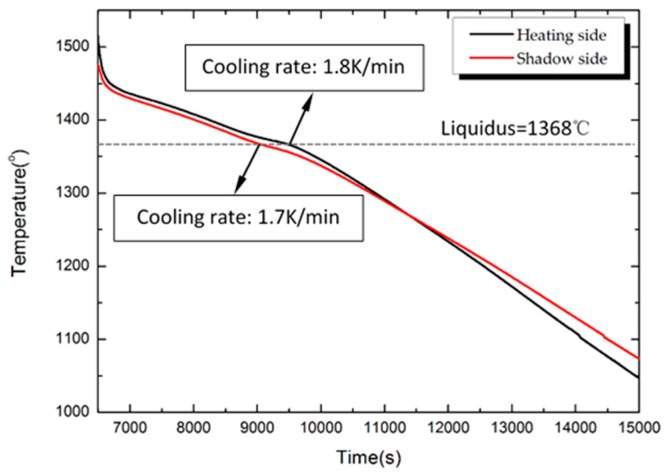
The temperature of the heating and the shadow side of the Ø19 mm rod during the solidification and cooling process (at a height of 190 mm from the chill plate).

The solidification experiments with superalloy CM 247 LC were carried out in a Bridgman furnace with a low temperature gradient (2.0 to 5.0 K/mm) and a withdrawal velocity of 1.0 mm/min. The temperature of the two annular upper and lower heating elements in the vacuum Bridgman furnace are shown in Figure 5. Heating temperature was controlled accurately by two annular upper and lower heating elements. The heating rate is 20 K/min in the range of 800 to 1450 °C during the heating process, and the shell mold heating temperature was held at 1450 °C when the specimens were moved from the hot zone to the cold zone. After mold cooling, the specimens were knocked out of the shell mold, and sand blasting was used to clean the surface. The characteristic freckle defects can be observed after macro-etching with metallographic microscope. The cooling temperature curve of the heating and the shadow side of the Ø19 mm casting rod (at a height of 190 mm from the chill plate) during directional solidification process is presented in Figure 6. As shown in Aune’s research [15], the solidus and liquidus of alloy CM247 LC are 1282 °C and 1368 °C, respectively. Therefore, the cooling rate of the heating side and the shadow side of the Ø19 mm casting rod are 1.8 K/min and 1.7 K/min, respectively.

### 2.4. Metallographic and Composition Analysis

A series of rods with varying diameters were directionally solidified in the cluster mold in an industrial Bridgman furnace. After cooling and being knocked out of the shell mold, the rods were cut off from the casting cluster and sand blasted to remove any residue on the surface. Macro-etching examination with a solution of 50 pct H_2_O_2_ and 50 pct HCl, and a micro-etching in a solution of 60 pct alcohol, 40 pct HCl, and 2 g Cu_2_Cl·2H_2_O was employed to determine whether and where freckles were present in the casting rods.

The microstructures of the freckles on the single crystal superalloy components were characterized by optical microscopy (OM) and scanning electron microscopy (SEM). The compositions in the matrix and freckle regions of the as-cast CM247 LC were determined by energy dispersive X-ray analysis (EDS).

## 3. Results

### 3.1. Freckle Occurrence on the External Surface

**Figure 7:**
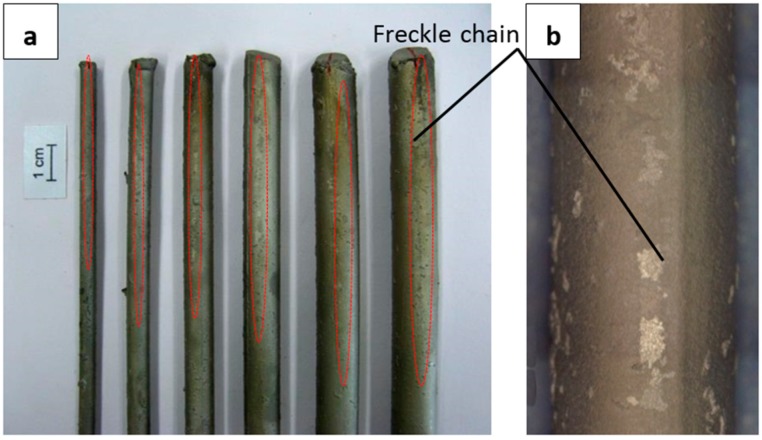
Freckle defects on CM247 LC casting rods with diameters of 5 mm, 7 mm, 9 mm, 11 mm, 13 mm, and 15 mm after directional solidification in a vacuum Bridgman furnace with a low withdraw rate of 1.0 mm/min. (**a**) freckles location; (**b**) magnification picture of freckles.

Figure 7a shows the CM247 LC rods with diameters of 5 mm, 7 mm, 9 mm, 11 mm, 13 mm, and 15 mm after macro-etching. The freckle chains (Figure 7b, in bright and gray colors) on the surface were parallel to the solidification direction. The freckles occurred on the external surface of all of the rods with cross sectional areas of 20 mm^2^, 39 mm^2^, 64 mm^2^, 95 mm^2^, 133 mm^2^, and 177 mm^2^. Freckle chains are marked by circles in Figure 7a, and the initial position of the freckles (the height from the chill) were 189 mm, 164 mm, 172 mm, 156 mm, 158 mm, and 155 mm, respectively. The initial position of freckles in the 15 mm diameter rod was lower than the other rods with smaller cross sectional areas.

**Figure 8:**
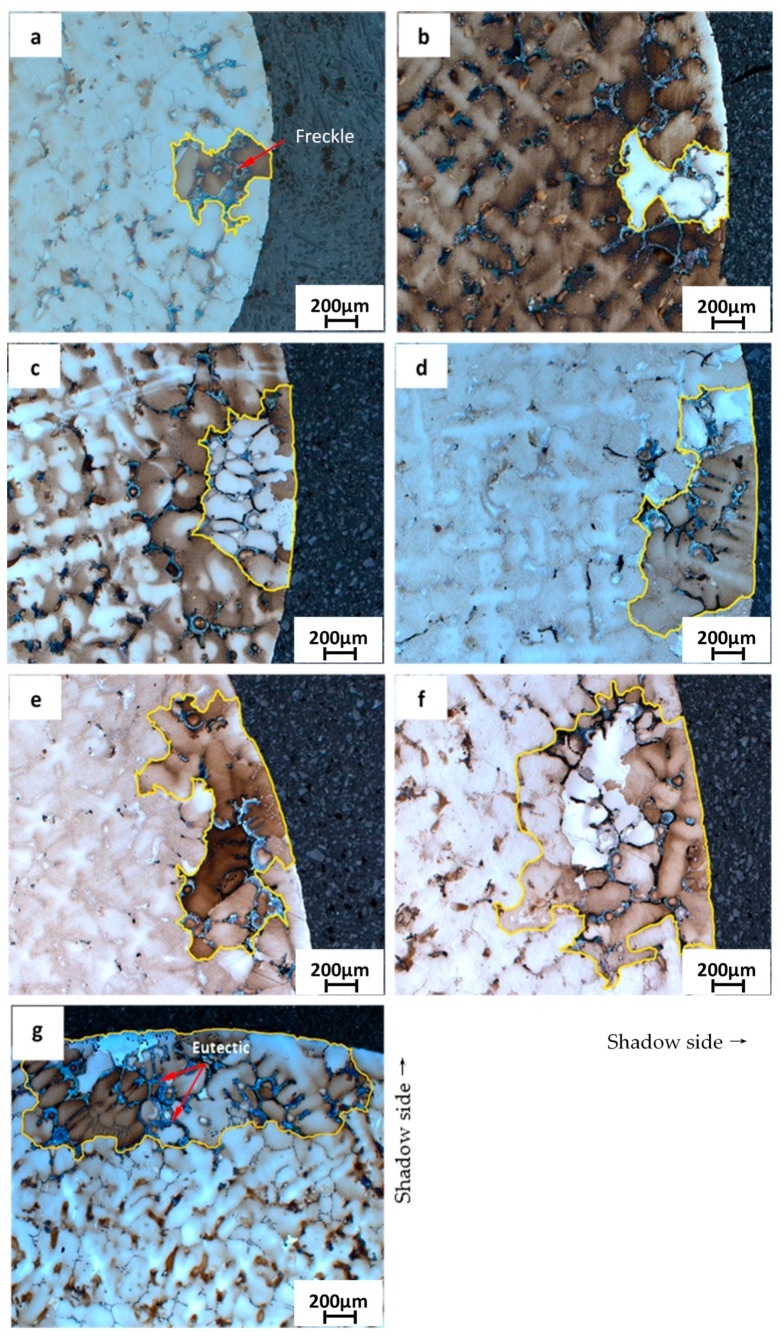
Freckle observed under metallurgical microscopy on the cross section of CM247 LC casting rods (at a height of 210 mm from the cooling chill) with diameters of (**a**) 5 mm; (**b**) 7 mm; (**c**) 9 mm; (**d**) 11 mm; (**e**) 13 mm; (**f**) 15 mm; and (**g**) 19 mm.

The cross sections containing freckle chains on the top of the CM247 LC rods (a height of 210 mm from the cooling chill) with diameters of 5 mm, 7 mm, 9 mm, 11 mm, 13 mm, 15 mm, and 19 mm are marked by a yellow frame in Figure 8a–g under metallurgical microscopy. As shown in Figure 7b and Figure 8, the freckle defect is a linear trail of equiaxed grains which have different orientations, and the freckle area is reflected by different colors (in bright and gray colors), compared with the matrix area which has a single crystal structure. It appears as dark and white spots in the cross sections of the metallographs (a) to (g) in Figure 8. The area of freckles increases with increasing cross sectional area of the specimens from 20 mm^2^ to 283 mm^2^. Meanwhile, the γ/γ′ eutectic phase is depicted by arrows in the freckle area, and it can be seen that the freckle area has a higher fraction of γ/γ′ eutectic than the matrix.

### 3.2. Freckle Occurrence on Multi-Interfaces

**Figure 9:**
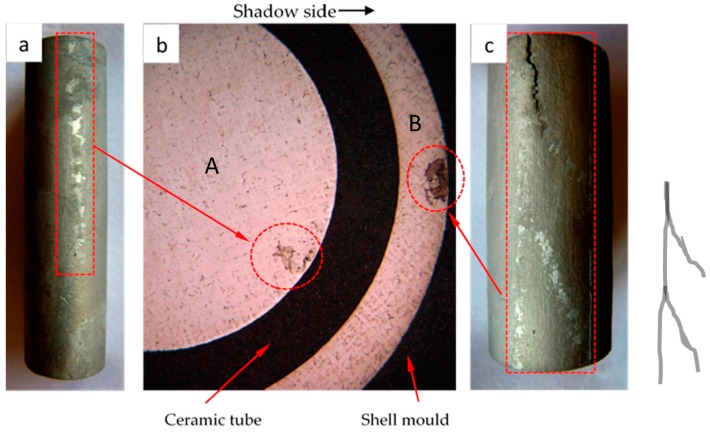
Freckle chain occurs on the: (**a**) internal surface and (**b**) cross section of specimen; (**c**) external surface of CM247 LC.

The CM247 LC casting specimen with multi-interfaces (Specimen No. 8 with an internal diameter of 12 mm and an external diameter of 19 mm) is shown in Figure 9. After removing the shell mold material, freckle defects were observed on the cross section of the specimen. Seen in Figure 9b, the black area between the internal and external superalloy is the ceramic tube, meanwhile, the black area near the surface of the external superalloy is the shell mold. The internal and external freckle chains on the vertical direction of the specimen are shown in Figure 9a,c, which were separated from Specimen No. 8. The freckles occurred not only on the external surface but also on the internal interface. One freckle chain was observed on the surface of the inner superalloy A, meanwhile, three freckle chains on the outer superalloy B were observed at the low height position from the cooling chill, and three freckle chains converged to one chain at the higher position from the cooling chill. Figure 9b indicates that freckles near the inner-interface and outer-interface occurred on the shadow side, which had a lower cooling rate.

**Figure 10:**
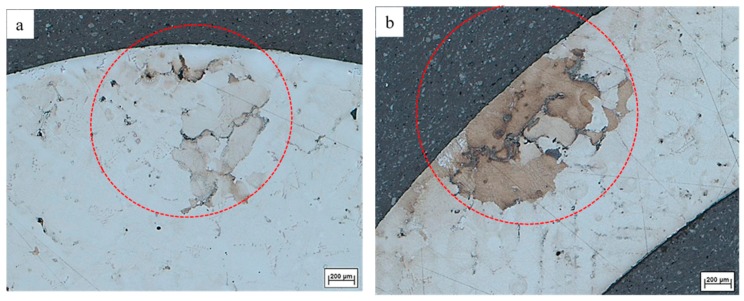
Magnified photos of freckles near the multi-interfaces (cross section of superalloy CM247 LC in Figure 6) on (**a**) the interface of the inner superalloy A and (**b**) the interface of the outer superalloy B.

Figure 10a shows the freckles on the cross sectional surface of the inner superalloy A and Figure 10b shows the freckles on the surface of the outer superalloy B. The depth and width of the freckle chain on the surface of the outer superalloy are 1569 µm and 833 µm, respectively. Moreover, the depth and width of the freckle chain on the surface of the inner superalloy is 1114 µm and 1236 µm, respectively. The area of the freckles on the outer and inner superalloy is 1.01 mm^2^ and 0.89 mm^2^, respectively. It indicates that the side and area of the freckle chain on the outer superalloy B is greater than that on the inner superalloy A.

**Figure 11:**
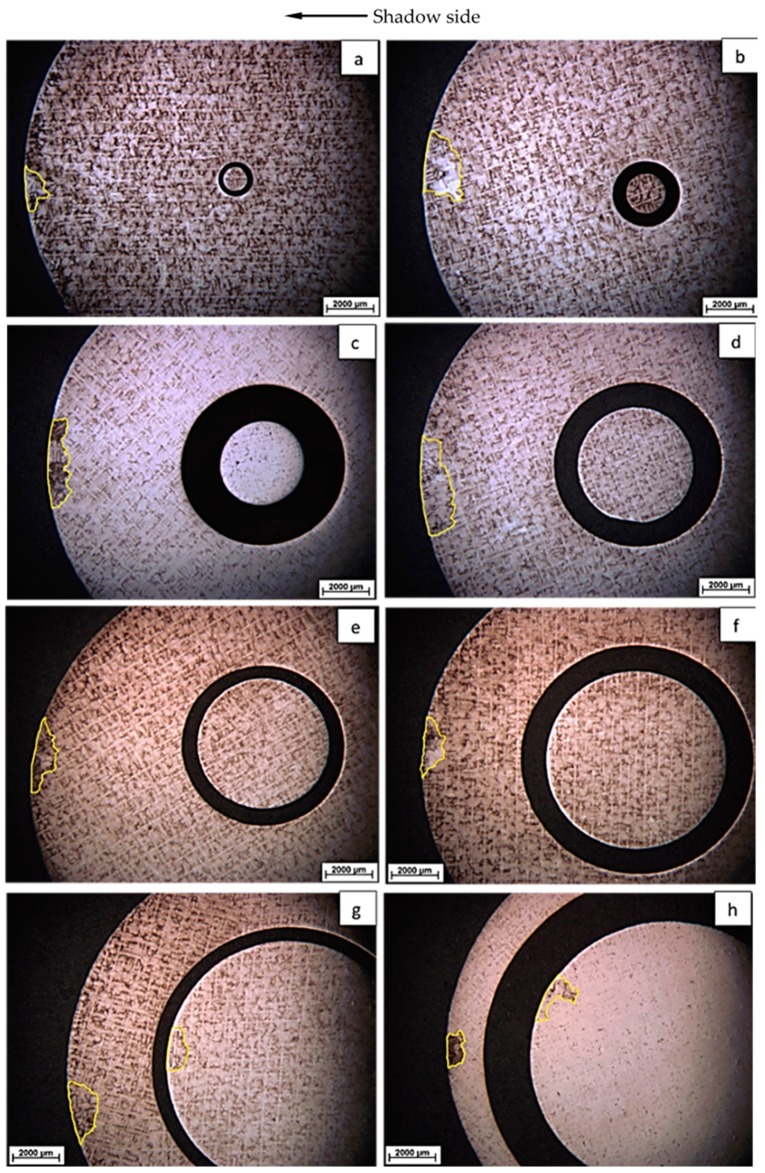
Freckles on the specimens with various diameters of ceramic tubes inside: (**a**) 1 mm; (**b**) 2 mm; (**c**) 4 mm; (**d**) 5 mm; (**e**) 6 mm; (**f**) 8 mm; (**g**) 11 mm; and (**h**) 12 mm.

The cross sections at a height of 190 mm from the cooling chill, with various diameters of ceramic tubes, are shown in Figure 11a–h and are as follows. Freckle defects were found both near the inner-interface and outer-interface in the specimens with 11 mm and 12 mm diameters, shown in Figure 11 and Table 4. The freckle defects were observed on the shadow side of the outer-interface surface ceramic tubes with diameter of 1 mm, 2 mm, 4 mm, 5 mm, 6 mm, 8 mm, 11 mm, and 12 mm inside of the specimens.

**Figure 12:**
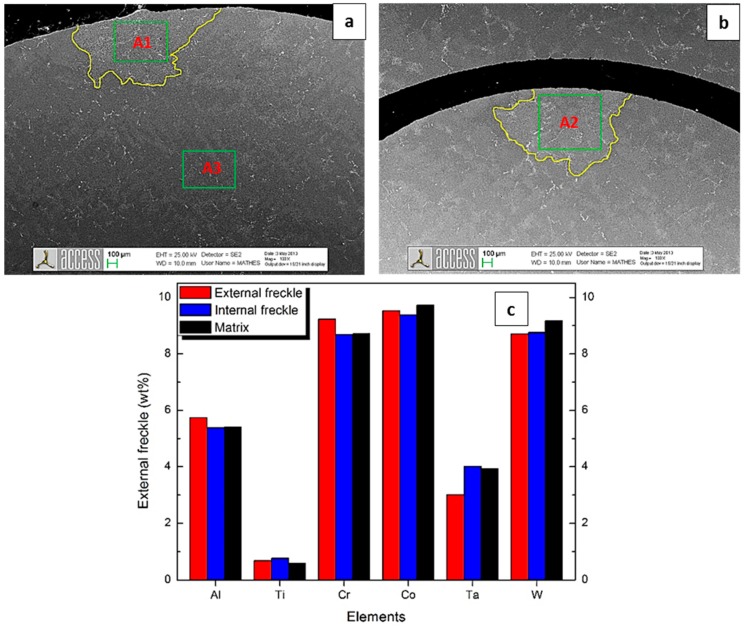
Composition (in wt %) in the matrix and freckle area of as-cast CM 247 LC with a 11 mm diameter ceramic tube inside, by SEM with EDS: (**a**) Inspection zone A1 is the external freckle area, Inspection zone A3 is the matrix area; (**b**) Inspection zone A2 is the internal freckle area; (**c**) EDS results.

The chemical composition of freckles on the external surface (Inspection zone A1), freckles on the internal surface (Inspection zone A2), and in the matrix (Inspection zone A3) were investigated by SEM with energy dispersive X-ray analysis (EDS), shown in Figure 9a,b. The results of the chemical composition in zones A1, A2, and A3 are shown in Figure 12c.

## 4. Discussion

### 4.1. Influence of Height from Cooling Chill

**Figure 13:**
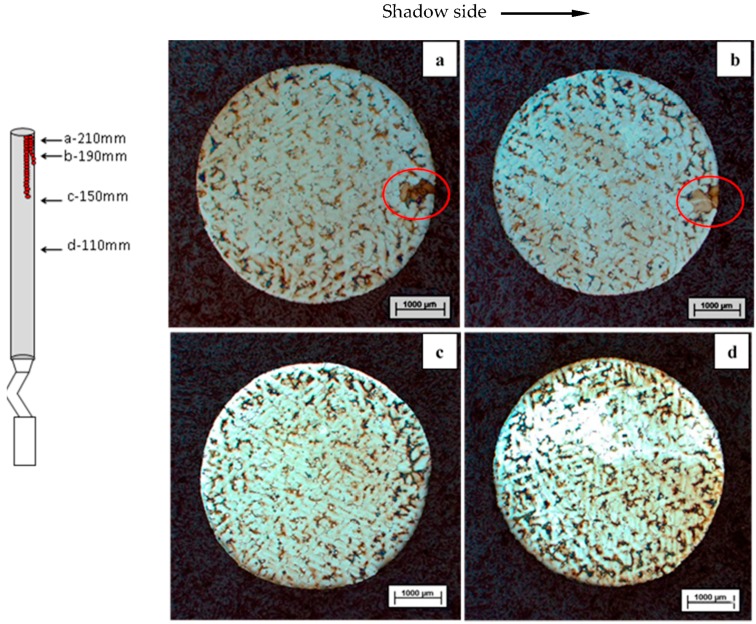
Cross sections of CM247 LC at different heights from the cooling chill (with a 5 mm diameter), freckles were observed above a height of 150 mm from the chill plate: (**a**) height of 210 mm; (**b**) height of 190 mm; (**c**) height of 150 mm; (**d**) height of 110 mm.

Based on the present experiments and past research [16], freckles do not occur on the bottom of rods immediately, but start from a particular height from the cooling chill. Metallographic analysis was carried out by cutting the samples at heights of 110 mm, 150 mm, 190 mm, and 210 mm from the cooling chill, which is perpendicular to the solidification direction, shown in Figure 13. Freckles were observed on the cross section a (height of 210 mm) and cross section b (height of 190 mm) on the shadow side in the furnace, and no freckle was observed on the section a (height of 150 mm) and section b (height of 110 mm).

**Figure 14:**
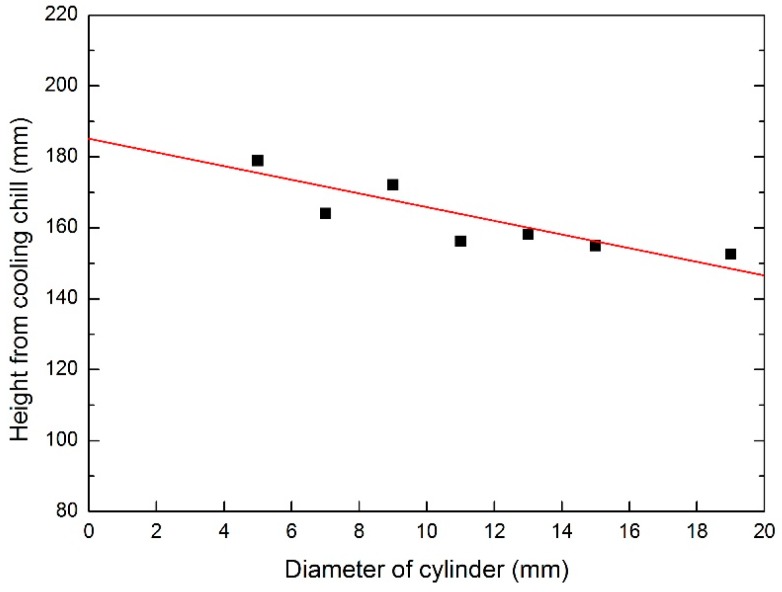
Initiation position of the freckle chain in various diameters.

Moreover, the height of initiation position of the freckle chain reduces when the diameter of rod increases. As shown in Figure 14, the height of freckle initiation position was 178.9 mm with a diameter of 5 mm, but it decreased to 152.6 mm with a diameter of 19 mm. In the same solidification condition, large diameter specimens have a higher probability and less incubation height of freckle formation. It is indicated that the initiation position and average size of the freckle chain are relative with rod diameter in the same solidification process.

**Figure 15:**
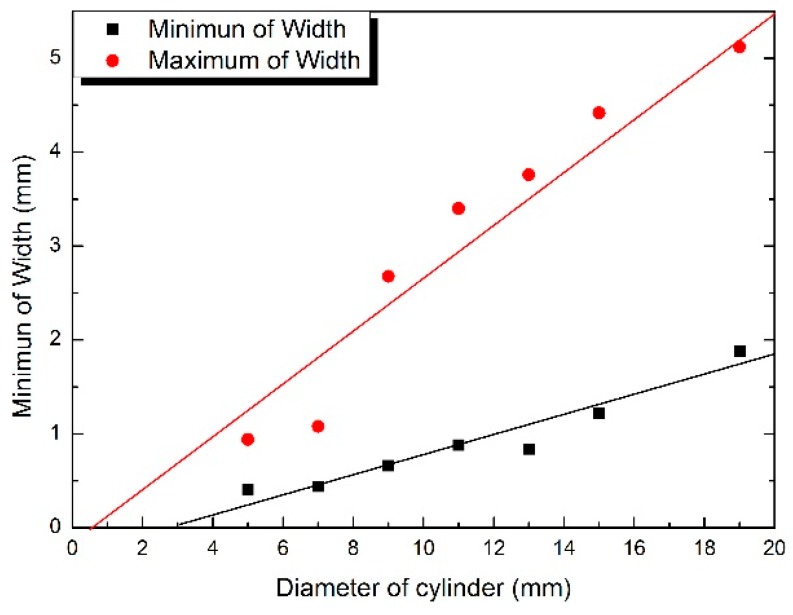
Statistics of the maximum and minimum width of freckle chains with incremental diameter of the specimens.

In Figure 15, the maximum and minimum width of freckle chains on the specimens was measured using a metalloscope. It indicated that the maximum and minimum width of freckle chain increases when the diameter of the rod increases. The maximum width of the freckle chain is 0.94 mm when the diameter is 5 mm, but it rises to 5.12 mm when the diameter is 19 mm. The minimum width of the freckle chain is 0.40 mm when the diameter is 5 mm, and it rises to 1.90 mm when the diameter is 19 mm.

**Figure 16:**
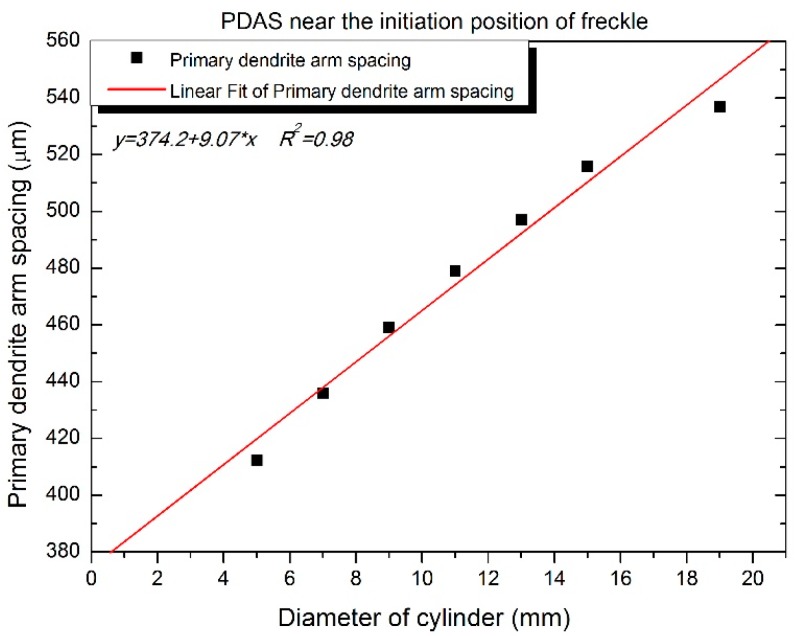
Experimental date and the fitting line of the primary dendrite arm spacing (*λ*_1_) at the height of the freckle initiation position with diameters of 5 mm, 7 mm, 9 mm, 11 mm, 13 mm, 15 mm, and 19 mm.

Specimens were cut-up as cross sections near the initiation position, and the primary dendrite arm spacing (PDAS, *λ*_1_) was measured and calculated using image processing software. It indicated that the primary dendrite arm spacing of incremental diameter (incremental cross sectional area) follows the linear relationship in Figure 16. The dot is the experimental date and the red line indicates linear fitting of the primary dendrite arm spacing as a function of the diameter. The fitting equation is y *=* 374.2 + 9.07x. The Rayleigh number developed by Sarrazin and Hellawell could be written as follows [17]:

(1) $$R_{a}=\frac{g\frac{d\rho }{dT}}{\eta D_{T}}\times G\lambda_{1}^{4}$$

As the primary dendrite arm spacing (*λ*_1_) increases, the Rayleigh number (*R_a_*) increases. The tendency of freckle formation would be promoted when *R_a_* increases. The result in Figure 16 agrees with the Rayleigh number criterion that freckle chain occurs preferentially in the large size rods because it has a greater *λ*_1_, which means that freckle chain has more space and less resistance from liquid in the interdendritic area in the mushy zone.

### 4.2. Influence of Cross Sectional Area

**Figure 17:**
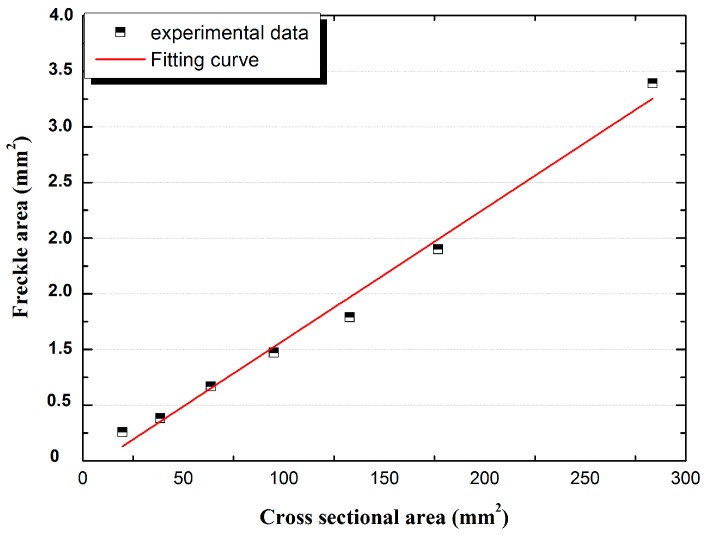
Freckle area and fitting curve on the top of CM247 LC rods for various cross sectional areas from 20 to 284 mm^2^.

The area and area percentage of freckles on the cross sections shown in Figure 8a–f were measured. As shown in Figure 17, the area of freckles on the top were 0.26 mm^2^, 0.38 mm^2^, 0.67 mm^2^, 0.97 mm^2^, 1.29 mm^2^, 1.90 mm^2^, and 3.39 mm^2^ which follows the linear relationship with the cross sectional area of 20 mm^2^, 39 mm^2^, 64 mm^2^, 95 mm^2^, 133 mm^2^, 177 mm^2^, and 284 mm^2^, respectively. The area of freckles increases when the cross sectional area increases.

**Figure 18:**
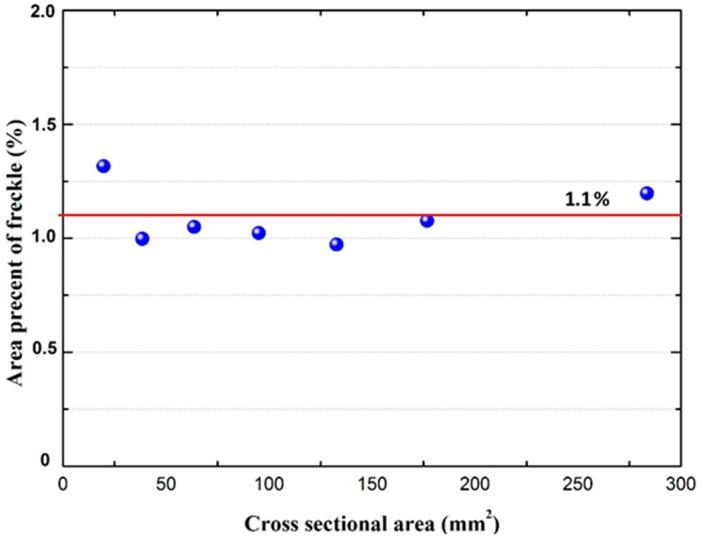
Freckle area percentage and fitting curve on the top of CM247 LC rods for various cross sectional areas from 20 to 284 mm^2^. The average value is 1.1% from this figure.

As shown in Figure 16, when the primary dendrite arm spacing (*λ*_1_) increases, the Rayleigh number (*R_a_*) also increases, which has a higher tendency of defect formation. However, there is no significant change in the area percentage of freckles when the cross sectional area increases; it maintains a constant level, and the average value of the area percentage of freckles was 1.1% of the cross sectional area, shown in Figure 18.

### 4.3. Interface Effects of Freckle Formation

**Figure 19:**
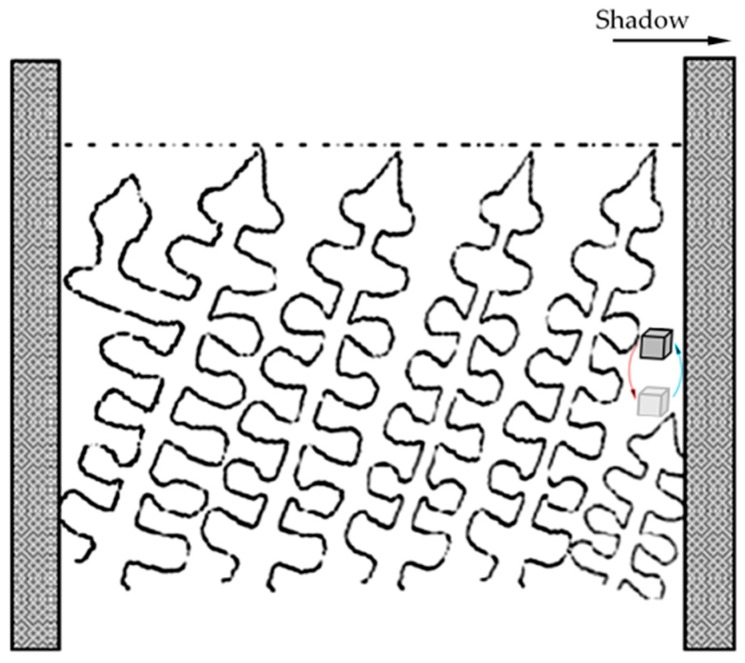
Schematic diagram of the inclination of the primary dendrites with respect to the mold wall. Micro-segregation causes density inversion near the interface of the mold wall and casting on the shadow side.

In the present work, ceramic cores inside of the shell mold created new interfaces in the casted parts. As the results in Figure 8 and Figure 11 show, freckle defects were observed only on the shadow side which faced the center rod, however, no freckle defects were present on the heating side which faced the heating ring in this work. The shadow-heating effect leads to the inclination of primary dendrites during directional solidification. Brewster G. and Dong H.B. indicated that the increased micro-segregation at the surface is the inclination of the primary dendrite stem with respect to the mold wall [18,19]. As shown in Figure 19, extensive branching of the secondary arms on the diverging side, and the subsequent formation of tertiary arms to become new primary stalks, occurs to fill the open body of liquid at the groove at the mold wall (near the left side of the mold wall in Figure 19). Consequently, dendrite inclination could potentially lead to the formation of surface eutectic. Meanwhile, micro-segregation in the mushy zone caused density inversion near the interface of the mold wall and casting on the shadow side.

**Figure 20:**
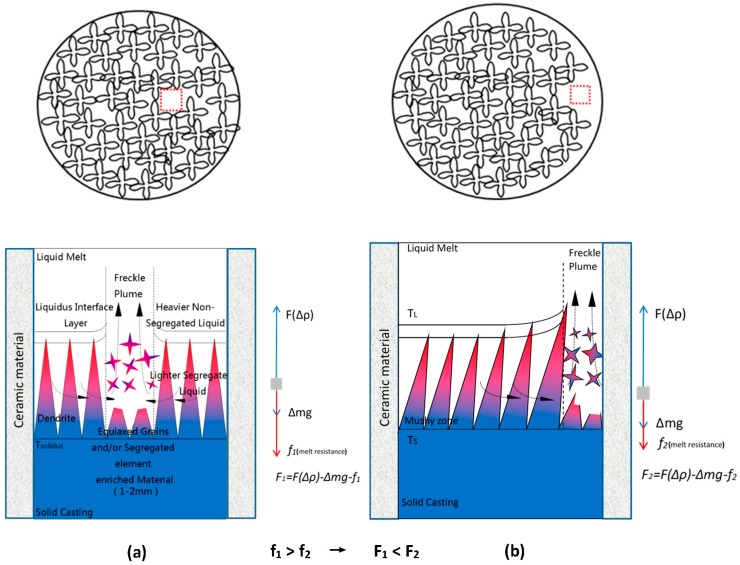
Schematic diagram of freckle formation (**a**) inside of superalloy and (**b**) near the interfaces of superalloys and ceramic materials.

As shown in Figure 11, freckle defects occurred near both the internal and external interfaces if the height and specimen section size were in the range of freckle tendency. In the previous work [20,21,22,23,24,25,26], freckle chains were observed inside superalloy parts, especially in the center to mid-radius of VAR/ESR ingots and superalloy billets. A model sketch of freckle formation and the associated fluid flow pattern for internal freckles was applied in Figure 20a. Freckle defects were caused by fluid flow named thermosolutal convection, originating in the interdendritic region of the mushy zone during solidification. The Rayleigh number can be used to predict freckle formation with good precision, and combines alloy effects and solidification conditions. In Figure 20a, the freckle chain is surrounded by dendrites, so the resistance of fluid flow comes from the surrounding dendrites forest in the mushy zone. This kind of flow was driven by the density inversion occurring as a result of micro-segregation, as shown in Figure 19. The fluid flow rate in the interdendritic region can be obtained from Darcy’s law, and the driving force (*F*_1_) of the fluid flow came from the density difference and gravity. The permeability of the mushy zone is a function of *λ*_1_, *λ*_2_, and the liquid fraction *f_L_*, so the permeability where freckles occurred inside of superalloy parts is defined as [27,28,29]:

(2) $$\Pi =\Pi \left(f_{L}\right)*\Pi \left(\lambda_{1}*\lambda_{2}\right)=K_{p}*f_{L}^{a}*\lambda_{1}^{b}*\lambda_{2}^{c}$$

where Π is the permeability, *λ*_2_ is the second dendrite arm space (SDAS), and $K_{p}$ is a constant.

Therefore, the Rayleigh number could be written as:

(3) $$Ra=g*\frac{∆\rho *\Pi \left(f_{L}\right)}{\upsilon *f_{L}}*\frac{\Pi \left(\lambda_{1}*\lambda_{2}\right)}{R}*\sin\alpha *\cos\left(\emptyset +\alpha \right)\;$$

As shown in Figure 20a, where ∆*F*(*ρ*) is the buoyancy force of the density difference in the mushy zone, ∆mg is the dead weight of the fluid, and *f*_1_ is the resistance of fluid flow from the surrounding the dendrites forest. Hence, the driving force *F*_1_ is defined as:

(4) $$F_{1}=∆F\left(\rho \right)-∆\mathrm{mg}-f_{1}$$

A new sketch of freckle formation and associated fluid flow pattern for freckles near the interface was applied in Figure 20b. The permeability $\Pi_{1}$ where freckles occurred near both of the internal and external interfaces of superalloy parts is defined as:

(5) $$\Pi_{1}=A*K_{p}*f_{L}^{a}*\lambda_{1}^{b}*\lambda_{2}^{c}$$

where ∆*F*(*ρ*) is the buoyancy force of the density difference in the mushy zone, ∆mg is the dead weight of the fluid, and *f*_2_ is the resistance of fluid flow from the surrounding the dendrites forest. So, the driving force *F*_2_ in this condition is defined as:

(6) $$F_{2}=∆F\left(\rho \right)-∆\mathrm{mg}-f_{2}$$

where *A* is a constant related to the surrounding dendrite forest, with a value range of 1>A>0. Equation (5) is developed from Equation (2) based on the location of freckle occurrence. It is clear that the permeability $\Pi_{1}$ is less than Π, therefore, $f_{2}<f_{1},\;F_{2}\;>F_{1}$, and it has more freckle tendency near the interface rather than inside the parts. Supported by this work, freckles are observed near the interfaces in most situations. The liquid flow easily moved because the flow was not completely surrounded by dendrites; more than half of the space was instead a relatively smooth surface of ceramic materials. The resistance of the ceramic materials’ surface is less than in the interdendritic region, which was completely surrounded by dendrites. The flow of metal liquid near the interfaces was stronger than in the interdendritic region in the mushy zone.

## 5. Conclusions

The results indicated an interesting phenomenon, even though in the invariable casting condition and superalloy components, most of the freckle chains were observed on the shadow side and near the interface of the superalloys and ceramic materials. In this work, freckles occurred on the external surface near the interface of the superalloys and shell mold wall, or occurred on the internal surface near the interface of the superalloys and the wall of ceramic materials inside of the castings. Furthermore, the initiation position of freckle formation occurred with some regularity, and the height of the freckle initiation position from the cooling copper chill reduced from small cross section to large cross section in a series of rods with varying diameters (from Ø5 mm to 19 mm in diameter). This new phenomenon provides a reminder that freckles are more likely to occur near the interfaces if ceramic cores are used for the hollow turbine blade process.

In the present work, the specimens with multi-interfaces were directional solidified. Freckle formation near the interface of multi-interfaces of the superalloy was investigated. The conclusions are as follows:

1. Freckle defects do not only occur on the external surface of Ni-based superalloys castings, but also on the internal surface, near the interface of superalloys and ceramic materials.
2. The height of the initiation position of the freckle chain reduces when the rod diameter increases. Meanwhile, the size of the freckle chain increases when rod diameter increases.
3. Freckle area on the rod follows a linear relationship with the cross sectional area. However, there is no significant change of the area percentage of freckles, and the average value is 1.1% of the cross sectional area.
4. Freckle defects occur on the large size internal superalloy parts (Diameter of 11 mm and 12 mm), but not on the small sizes (1 to 8 mm).

## Figures and Tables

**Table 1:** Nominal composition of the investigated superalloy CM247 LC (in wt %).

Alloy	Cr	Co	Mo	W	Ta	Al	Ti	Hf	B	C	Zr	Ni
CM247 LC	8.1	9.2	0.5	9.5	3.2	5.6	0.7	1.4	0.015	0.07	0.015	Bal.

**Table 2:** Solid specimens in diameter (mm) and cross sectional area (mm^2^).

Specimen No.	1	2	3	4	5	6	7
Diameter (mm)	5	7	9	11	13	15	19
Cross Sectional area (mm^2^)	20	39	64	95	133	177	283

**Table 3:** Specimens with multi-interfacial features in diameter (mm) and cross sectional area (mm^2^).

Specimen No.	1	2	3	4	5	6	7	8
External diameter (mm)	19	19	19	19	19	19	19	19
Internal diameter (mm)	1	2	4	5	6	8	11	12
Internal cross sectional area (mm^2^)	0.8	3	13	20	28	50	95	113

**Table 4:** Freckle statistics of a series of rod specimens with multi-interfacial features in the diameter.

Specimen No.	1	2	3	4	5	6	7	8
External diameter (mm)	19	19	19	19	19	19	19	19
Freckle?	YES	YES	YES	YES	YES	YES	YES	YES
Internal diameter (mm)	1	2	4	5	6	8	11	12
Freckle?	NO	NO	NO	NO	NO	NO	YES	YES
